# Estrogenic and Antioxidant Activities of *Pterocarpus soyauxii* (Fabaceae) Heartwood Aqueous Extract in Bilateral Oophorectomized Wistar Rat

**DOI:** 10.1155/2021/6759000

**Published:** 2021-09-30

**Authors:** Yolande Sandrine Mengue Ngadena, Pascal Emmanuel Owona, Michel Noubom, Michel Arnaud Mbock, Lohik MbolangNguegan, Madeleine Chantal Ngoungouré, Rodrigue Ngapout Fifen, Ronald Bidingha A Goufani, Rivaldo Bernes Kahou Tadah, Danielle Claude Bilanda, Pierre Kamtchouing, Paul Désiré Dzeufiet Djomeni

**Affiliations:** ^1^Department of Psychology, Faculty of Arts, Letters and Social Science, University of Yaoundé 1, P.O. Box 812, Yaoundé, Cameroon; ^2^Department of Animal Biology and Physiology, Laboratory of Animal Physiology, Faculty of Science, University of Yaoundé 1, P.O. Box 812, Yaoundé, Cameroon; ^3^Department of Biological Sciences, Faculty de Medicine and Pharmaceutical Sciences, University of Dschang, P.O. Box, 67, Dschang, Cameroon

## Abstract

Phytoestrogens are used to ease postmenopausal symptoms, a property probably due to estrogenic and antioxidant effects. *Pterocarpus soyauxii* (*P*. *soyauxii*) is empirically used in Cameroon to treat among others primary and secondary amenorrhea. The aim of this study is to evaluate estrogenic and antioxidant activities of *P*. *soyauxii* heartwood aqueous extract in bilateral oophorectomized Wistar rats. Firstly, a characterization of the extract was carried out. For that, flavonoids, phenols, and tannins levels in *P*. *soyauxii* extract were evaluated by colorimetric assays and UHPLC-MS analysis was realized. *In vitro* antioxidant analysis of *P*. *soyauxii* was conducted using DPPH, ABTS, and FRAP assays. Secondly, 2 sets of pharmacologic tests were carried out. The results revealed that *P*. *soyauxii* aqueous extract contains, respectively, 229.42 ± 3.62 mg EAG/g, 63.42 ± 2.16 mg EQ/g, and 27.88 ± 0.23 mg ETA/g of polyphenols, flavonoids, and tannins. UHPLC-MS enabled identifying seven components including mono(2-ethylhexyl) phthalate, cembrene, 3′,5′-dimethoxy-4-stilbenol, and linoleic acid. DPPH, ABTS, and FRAP assays revealed that *P*. *soyauxii* extract possessed a high antioxidant activity with IC_50_ value of 730.20 *µ*g/mL, 892.90 *µ*g/mL, and 765.75 mEAG/g of extract, respectively. In the uterotrophic assay, *P*. *soyauxii* extract induced significant increase of fresh uterine weight, uterine and vaginal epithelial size, and mammary glands differentiation compared to Ovx control. In the postmenopausal model, compared to the sham control, vagina and uterine dystrophies were observed in Ovx rats treated with distilled water. *P*. *soyauxii* aqueous extract expressed estrogenic-like effects on vagina and did not affect uterine epithelial height compared with vehicle groups. On the back of these vaginotrophic effects, the extract displayed antiatherogenic properties by reducing (*p* < 0.001) AI and LDL cholesterol level as compared to Ovx control group. The extract at 200 mg/kg significantly prevented the increase of MDA (*p* < 0.01) level and decreased nitrites (*p* < 0.001) and GSH (*p* < 0.01) levels compared to Ovx rats. These beneficial effects are related at least in part to the presence of compound such as mono(2-ethylhexyl) phthalate, 3′,5′-dimethoxy-4-stilbenol, and linoleic acid. Overall, *P*. *soyauxii* aqueous extract exhibits estrogenic and antioxidant effects which can inhibit postmenopausal symptoms by providing vaginal stratification, improving lipid profile and insulin sensitivity, and reducing oxidative stress without side effects on the endometrium and mammary gland in 84-day Ovx rats.

## 1. Introduction

Menopause is a physiological state characterized by hypoestrogenism and lead to complications including hot flashes, tachycardia, vaginal dryness, urogenital atrophy, high visceral fat, increase of body weight, cardiovascular diseases, and disruption in sex steroid feedback on gonadotropin secretion [1]. The associated decline in estrogen-related antioxidant power results in the rapid development of menopausal symptoms such as cardiovascular diseases [2]. Oxidative stress is generated by an imbalance between the production of reactive oxygen species (ROS) and antioxidant defense system [3]. It is related to many risk factors of cardiovascular diseases such as obesity, atherosclerosis, insulin resistance, hypercholesterolemia, endothelial dysfunction, and vascular inflammation [4–7]. Hormone replacement therapy (HRT) is the main line for prevention and treatment of cardiovascular diseases in postmenopausal women. Despite its many benefits, HRT has adverse side effects such as breast and endometrial cancers [8]. People also use vitamins *E* and C to manage oxidative stress in postmenopausal symptoms [9]. Nevertheless, despite their protective effects, they lose their antioxidant power through oxidation [10]. In order to cope with these adverse side effects, research has focused on alternatives to HRT. The use of phytoestrogens attracted researchers over the last few years because of their antioxidant and preventive effects on such chronic diseases like cardiovascular diseases [11]. Indeed, they possess estrogenic-like activity and provide effective and secure alternative to HRT. Several medicinal plants are used empirically to cope with primary health problems and their estrogenic activity. Among them, *Erythrina poeppigiana*, *Rheum rhaponticum*, and *Anthocleista schweinfurthii* are reported to mimic estrogen activities in menopausal conditions [12–14]. Nevertheless, phytoestrogens are reported to induce gastrointestinal and endometrial hyperplasia side effects [15]; thus, additional preclinical and clinical studies are required to establish the safety profiles of plant extract.

*Pterocarpus soyauxii* Taub which is the subject of this work belongs to Fabaceae known as the African Padauk. Existent in Cameroon, *Pterocarpus soyauxii* (*P*. *soyauxii*) also returned to Nigeria, Gabon, and the Democratic Republic of Congo [16]. The wood and stem bark of *P*. *soyauxii* are empirically used to treat hypertension and diabetes. Ethnomedically, *Pterocarpus* species are used to treat urogenital pathologies and infertility. Moreover, pterostilbene identified in the extract of *P*. *soyauxii* has been reported to exhibit estrogenic activities *in vitro* [17, 18]. Traditional healers of the Center Region of Cameroon assert that heartwood *P*. *soyauxii* maceration is used to treat primary and secondary amenorrhea and some menopausal symptoms. The main objective of the present study was to evaluate estrogenic and antioxidant activities of the aqueous extract of the heartwood of *P*. *soyauxii* in bilateral oophorectomized Wistar rats.

## 2. Materials and Methods

### 2.1. Chemicals

2,2′-Azinobis-3- ethylbenzothiazoline-6-sulfonic acid (ABTS), 1,1-diphenyl-2-picrylhydrazyl (DPPH), 2,4,6-tri (2-pyridyl)-s-triazine, potassium persulfate, and ascorbic acid were purchased from Sigma Chemical Co. (St. Louis, MO, USA). Diazepam (Valium^®^ 10 mg) and 17-*β* estradiol valerate (Progynova^®^ 2 mg) were purchased from DELPHRAM (Lille, France).

### 2.2. Plant Material

The leaves and heartwood of *Pterocarpus soyauxii* Taub were harvested in Ngomedzap (Center Region, Cameroon) during the rainy season at 7 : 30 am on April 21, 2020. These leaves were authenticated at the National Herbarium of Cameroon (HNC-IRA) by comparison with the specimen of Thomas O. W8175 deposited under the voucher number 56984HNC.

#### 2.2.1. Extraction

The collected *Pterocarpus soyauxii* heartwood pieces were air dried at room temperature for 30 days. 70 g of the powder obtained was macerated in 2 L of distilled water for 48 hours at room temperature. The macerate obtained was filtered using Whatman number 3 filter paper and the filtrate obtained was evaporated in a rotary oven at 45°C. This process made it possible to obtain 5.6 g of crude extract with a yield of 8%.

#### 2.2.2. Determination of Study Doses

The recommendations of the traditional healer allowed for obtaining from 400 mL of macerate 1.12 g of crude extract after drying. The dose used in rats was determined by multiplying the HED by 6.2 according to the method described by Nair [19], resulting in a dose of approximately 100 mg/kg. This dose was divided by 2 and then multiplied successively by 2 and 4 to obtain the doses of 50, 200, and 400 mg/kg. The dose of 300 mg/kg was obtained by averaging the doses of 200 and 400 mg/kg.

#### 2.2.3. Phytochemical Analysis

The analysis of the phytochemical composition of the aqueous extract of the heartwood of *P*. *soyauxii* was carried out by using quantitative assays. Indeed, the levels of flavonoids, polyphenols, and tannins were evaluated according to Broadhurst and Jones [20], Singleton and Rossi [21], and Zhishen et al. [22], respectively.

#### 2.2.4. UHPLC-MS Analysis of P. soyauxii Extract

UHPLC-MS analysis was used to identify the phytochemical profile of *P*. *soyauxii* extract in an attempt of standardization. To obtain high-resolution mass spectra of extract, a spectrometer (QTOF Bruker, Germany) equipped with a Heated Electrospray Ionization (HESI) source was used. The spectrometer operates in positive mode (mass range: 100–1500, with a scan rate of 1.00 Hz) with automatic gain control to provide high-accuracy mass measurements within 0.40 ppm deviation using sodium formate as calibrant. Spray voltage of 4.5 kV and capillary temperature of 200°C were used for assays. Nitrogen was used as sheath gas (10 L/min). The spectrometer was attached to an Ultimate 3000 (Thermo Fisher, Germany) UHPLC system consisting of LC-pump, diode array detector (DAD) (*λ*: 190–600 nm), autosampler (injection volume 10 *µ*L), and column oven (40 °C). The separation was performed using a Synergi MAXRP 100A (50 × 2 mm, 2.5 *µ* particle size) with a H_2_O (+0.1% HCOOH) (A)/acetonitrile (+0.1% HCOOH) (B) gradient (flow rate 500 *µ*L/min, injection volume 5 *µ*L). The sample was analyzed using a gradient program as follows: 95% A isocratic for 1.5 min, linear gradient to 100% B over 6 min, after 100% B isocratic for 2 min, the system returned to its initial condition (90% A) within 1 min and was equilibrated for 1 min. Based on the mass of compounds previously identified in *Pterocarpus genus*, identification of compounds was performed.

### 2.3. Animal Material

Healthy female albino Wistar rats (8–10 weeks old) weighing 120–130 g were supplied by the production facility of the Animal Physiology Laboratory, University of Yaoundé 1 (Cameroon). All rats were housed in clean plastic cages at the room temperature (natural cycle). They had free access to tap water and soy-free rat chow. The composition of animal diet was corn (60%), bone flour (3%), peanuts (5%), wheat (10%), fish flour (20%), salt (1%), and vitamin complex (Olivitazol) (1%). All experiments were conducted in accordance with the principles and procedures of the European Union on Animal Care (CEE Council 86/609) guidelines adopted by the Cameroon Institutional National Ethic Committee, Ministry of Scientific Research and Technology Innovation (Reg. number FWA-IRD 0001954).

### 2.4. Determination in Vitro of Antioxidant Properties

#### 2.4.1. DPPH Radical Scavenging Assay

The DPPH free radical scavenging assay was carried out for the evaluation of the antioxidant activity. This assay measures the free radical scavenging capacity of the investigated extract. DPPH is a molecule containing a stable free radical. In the presence of an antioxidant, which can donate an electron to DPPH, the purple color typical for free DPPH radical decays and the absorbance change is measured at 517 nm. The antiradical activity of the plant extract was examined based on the scavenging effect of the stable DPPH free radical activity [23]. Briefly, 2 mL of DPPH (0.1 mM prepared in methanol) was introduced into a test tube containing 0.5 mL of extract (0.1 to 1 mg/mL). Then the mixture was stirred well for 5 min and incubated in the dark for 60 min at room temperature (20°C). For the control tube, methanol was used in place of the extract. The reference used was ascorbic acid at concentrations of 0.1 mg/mL to 1 mg/mL. A calibration curve was drawn from this reference. The antioxidant activity of the plant extract was expressed as a percentage inhibition following the relationship:

(1) $$\begin{array}{c} \text{\% inhibition}=\frac{\left(\text{OD control}-\text{OD sample}\right)}{\text{OD control}}\times 100. \end{array}$$

The IC_50_ value (*µ*g/mL) is the effective concentration at which DPPH radicals were scavenged by 50% and the value was obtained by interpolation from linear regression analysis.

#### 2.4.2. Determination of Ferric-Reducing Antioxidant Power (FRAP) Assay

The assessment of ferric-reducing antioxidant power (FRAP) was performed based on the ability of the tested substance to reduce ferric tripyridyl triazine (Fe III TPTZ) complex to ferrous form (intense blue color) at low pH by using a modified method of Benzie and Strin [24]. The solution of TPTZ (2,4,6-tri (2-pyridyl)-s-triazine) was obtained by diluting TPTZ (10 mM) in 10 ml of HCl (400 mM diluted with distilled water) and 10 mL of 10 mM iron chloride (FeCl_3_) solution was prepared in distilled water. FRAP reagent was obtained by mixing 100 mL of acetate buffer (pH 3.6) with 10 ml of TPTZ solution and 10 ml of iron chloride solution. In test tubes containing 2 mL of FRAP reagent, 75 *μ*L of sample (extract/catechin) was added and the mixture was stirred and incubated for 15 minutes. Optical densities were read at the wavelength of 593 nm against white.

#### 2.4.3. ABTS Radical Scavenging Assay

To determine ABTS radical scavenging assay, the method described by Re et al. [25] was used. Radical ABTS was obtained as follows: in an Erlenmeyer flask, 0.0384 g of ABTS and 0.00662 g of potassium persulfate (K_2_S_2_O_8_) were weighed and then 10 mL of distilled water was added. The mixture was then solubilized for 5 min and incubated for 16 h at room temperature (20°C) in the dark before use. For the actual analysis, the ABTS solution was diluted with ethanol to an absorbance of 1.3 (±0.02) at 734 nm and stable at 30°C (initial OD). Then in a test tube, 1.8 mL of this diluted ABTS solution and 0.2 mL of extract (1 mg/mL) were introduced and shaken well. The absorbance reading was taken at 734 nm and the values considered were those that remained stable at room temperature for approximately 1 minute. Ascorbic acid was used as the reference antioxidant at the same concentrations as extract. The results were expressed as a percentage of inhibition and calculated according to the following formula:

(2) $$\begin{array}{c} \text{\% inhibition}=\frac{\left(\text{OD control}-\text{OD sample}\right)}{\text{OD control}}\times 100. \end{array}$$

### 2.5. In Vivo Experimental Design

#### 2.5.1. Effect of 3-Day Treatment with P. soyauxii

This test was performed according to the protocol described by the OCDE [26]. Forty 8-to-10-week-old female Wistar rats were used. Thirty-five of these rats were ovariectomized using a dorsal approach and 5 more underwent simulation surgery and formed the sham-operated group. 14 days after ovariectomy, the ovariectomized animals (Ovx) were randomized and then divided into 7 groups of 5 animals each for oral administration of treatments for 3 days. The aqueous extract of *Pterocarpus soyauxii* heartwood was administered at doses of 50, 100, 200, 300, and 400 mg/kg and estradiol valerate (E_2_V) was administered at a dose of 1 mg/kg. A group of Ovx animals and the sham-operated group received distilled water at 10 mL/kg. At the end of the treatments, vaginal smears were carried out, and then the animals were sacrificed by decapitation under anaesthesia with diazepam and ketamine. The estrogen-dependent organs (uterus, vagina, and mammary gland) were removed and then fixed in 10% buffered formaldehyde solution for histological analysis. Before fixation, the uterus was weighed and part of it was homogenized in McEwen's solution for the evaluation of the level of uterine proteins. At last, the fresh uterine weight, uterine protein levels, and uterine and vaginal epithelial size as well as mammary gland alveolar and ductal differentiation were assessed.

#### 2.5.2. Evaluation of Pterocarpus soyauxii Activities in a Postmenopausal Model

Thirty-five rats were either sham-operated (sham) or bilaterally ovariectomized (Ovx) like in the first experiment. Eighty-four days later, animals were distributed in seven different groups (*n* = 5) and treated per *os* once daily for 28 consecutive days as follows: sham and an Ovx group received distilled water, the 4 others batches received, respectively, 1 mg/kg of E_2_V, and *P*. *soyauxii* aqueous extract at 100, 200, and 300 mg/kg. At day 21, insulin tolerance test was realized. Animals were weighted weekly. Twenty-four hours after the last administration (day 29) and following a 12 h of overnight fasting, animals were sacrificed under light anaesthesia. Blood samples were taken and centrifuged at 3500 g (15 min at 4°C) to obtain serum samples which were kept at −15°C for the determination of total cholesterol (TC), triglycerides (TG), and high-density cholesterol (HDL-C) levels. The concentration of low-density lipoproteins cholesterol (LDL-C) and very-low-density lipoproteins (VLDL) levels was calculated using the Friedewald equation [27]. Uterus, vagina, and aorta were dissected out and cleaned of all soft tissues. Prior to immersion-fixation of organs in the formaldehyde 10% buffered for histological analysis, they were weighted. A portion of aorta was homogenized in Mac even buffer to evaluate antioxidant parameters. Abdominal fat of each animal was weighted.

### 2.6. Vaginal Cellular Differentiation

Vaginal smears were carried out at the end of the experiment using an eyedropper containing 10 *µ*L of NaCl 0.9%, placed on ringed slides, fixed, and coloured with Papanicolaou method [28]. Cellular differentiation was observed under a light microscope at ×100 magnification.

### 2.7. Insulin Tolerance Test (ITT) in Ovariectomized Rats

Prior to an ITT test, rats were fasted for 12 h. A single dose of insulin at a dose of 2 UI/kg was injected *i*.*p.* into each rat. Blood samples were taken from tail veins and the blood sugar levels were measured using the Accu-chek reactive strips at 0 (just before the insulin injection), 10, 20, 30, and 60 min after injection of insulin.

### 2.8. Determination of Relative Weight of Organs

The relative fresh weight of uterus, abdominal fat, and aorta was calculated using the following formula according to [29]:

(3) $$\begin{array}{c} \text{Organ weight ratio}=\frac{\text{Uterus Weight}\left(\text{g}\right)}{\text{Body weight }\left(\text{g}\right)}\times 100\text{.} \end{array}$$

### 2.9. Assays for Lipid Profile

Serum total cholesterol (TC), triglycerides (TG), and HDL cholesterol (HDL-C) levels were assessed using commercial diagnostic kits Fortress, UK. The levels of LDL cholesterol (LDL-C) were assessed by using the following formula: LDL-Chol (mg/dL) = Chol - (TG/5) - HDL-Chol. VLDL cholesterol (VLDL-C) level was calculated with the following formula: TG/5 [27]. Atherogenic index (AI) was calculated as TC on HDL-C.

### 2.10. Oxidative Stress Parameters Investigation

Malondialdehyde (MDA) and reduced glutathione (GSH) in aorta homogenate were determined using the methods described by Wilbur et al. [30] and Ellman [31], respectively, while the nitrites content was determined using the method described by Green et al. [32].

### 2.11. Histomorphometric Analysis of Uterine, Vaginal, and Aorta Tissues

Aortic, uterine, vaginal, and mammary gland tissues after fixation (2 weeks) in formaldehyde 10% buffered were trimmed and dehydrated in alcohol of croissant gradient (70%, 80%, 90%, and 100% (3 baths)). After tissues were clarified in 2 baths of xylene (1h30 min per bath) and impregnated in liquid paraffin at 60°C (for 5 hours), uterine and vaginal epithelial sizes as well as intima and media heights were assessed from 5 *μ*m sections of paraffin-embedded and haematoxylin–eosin stained uterine and vaginal tissues. Epithelial sizes were assessed on microphotographies obtained by using a light microscope (Leitz wetzlar Germany 513) connected with a digital camera celestron 44421 linked to a computer where images were transferred.

### 2.12. Statistical Analysis

Data were expressed as mean ± standard error on mean. Statistical analysis was performed using one-way analysis of variance (ANOVA) followed by the Tukey post hoc test using GraphPad Prism 8.0.1. A value of *p* ˂ 0.05 was considered statistically significant.

## 3. Results

### 3.1. Phytochemical Analysis

The phytochemical analysis revealed that heartwood of *Pterocarpus soyauxii* extract contains, respectively, 229.42 ± 3.62 mg EAG/g, 63.42 ± 2.16 mg EQ/g, and 27.88 ± 0.23 mg ETA/g of polyphenols, flavonoids, and tannins (Table 1).

### 3.2. Identification of Compounds of P. soyauxii Extract by UHPLC-MS Analysis

**Figure 1:**
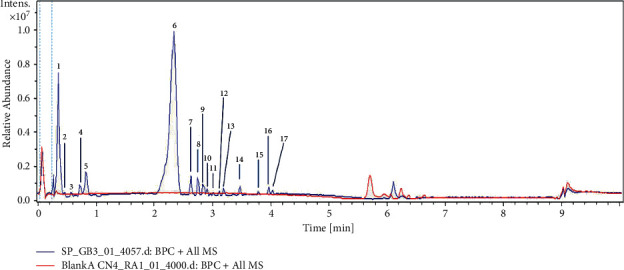
Chromatographic profile of *Pterocarpus soyauxii* aqueous extract.

Ultra-High-Performance Light Chromatography allowed the separation of components of the aqueous extract of *Pterocarpus soyauxii*. Ions mass spectrometry and a representative base peak chromatogram are shown in Figure 1 and Table 2. UHPLC-MS enabled identifying seven components: ambrial, 7-O-acetylformononetin, khrinone A, mono(2-ethylhexyl) phthalate, cembrene, 3′,5′-dimethoxy-4-stilbenol, and linoleic acid.

### 3.3. In Vitro Antioxidant Activity

According to the results obtained in Table 3, vitamin C exhibits greater anti-free radical activity than that of our extract. It had the smallest IC_50_ against the DPPH and ABTS radicals. The inhibitory concentration 50 of vitamin C was 24.56 *µ*g/mL while the aqueous extract of *P*. *soyauxii* was 730.20 *µ*g/mL for the DPPH radical while it was 37.75 for vitamin C against 892.90 for the extract of PS for the radical ABTS. The concentration of FRAP was 765.75 mEAG/g.

### 3.4. Effects of a 3-Day Treatment with P. soyauxii

#### 3.4.1. Effect on Relative Weight, Protein Level, and the Size of the Epithelium of Uterus

**Figure 2:**
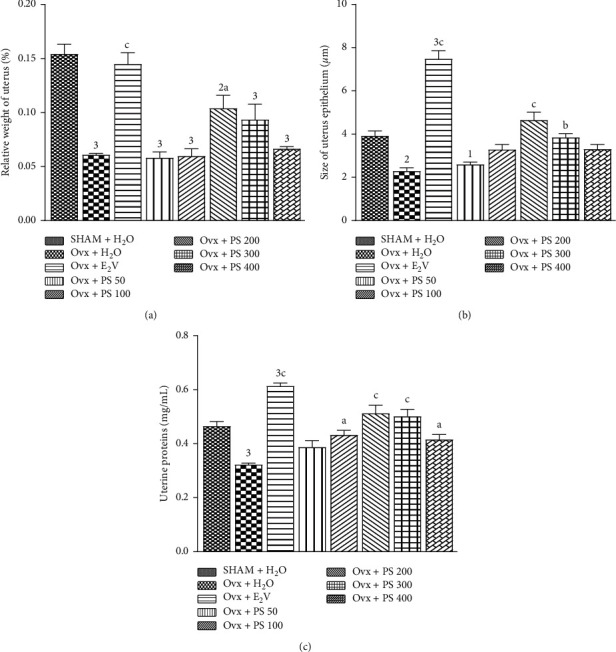
Effects of a 3-day treatment with *P*. *soyauxii* on relative weight of fresh uterus. (a) Size of uterine epithelium. (b) Total uterine protein levels. (c).^1^p < 0.05; ^2^*p* < 0.01; ^3^*p* < 0.001, significant difference compared to sham-operated control; ^a^*p* <0.05; ^b^*p* < 0.01; ^c^*p* < 0.001, significant difference compared to Ovx control rats. PS = *P*. *soyauxii*.

Figure 2 represents effects of 3-day treatment with aqueous extract of *P*. *soyauxii* heartwood on the relative weight of the uterus (Figure 2(a)), the size of the uterine epithelium (Figure 2(b)), and uterine protein level (Figure 2(c)). The extract induced a significant increase in the relative weight of the uterus and the size of the uterine epithelium at the doses of 200 and 300 mg/kg with respective probabilities of *p* < 0.001 and *p* < 0.01 as compared to the ovariectomized animals treated with distilled water. Figure 2(c) also shows that the extract induced a significant increase in uterine protein levels (*p* < 0.05) at doses of 100 and 400 mg/kg. The doses of 200 and 300 mg/kg also significantly increased this uterine protein level (*p* < 0.001) in comparison to animals in the Ovx control.

#### 3.4.2. Effect on the Size of the Vagina Epithelium

**Figure 3:**
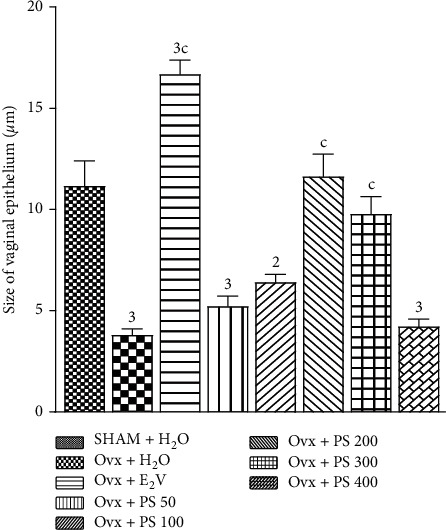
Effect of a 3-day treatment with *P*. *soyauxii* on vaginal epithelium. ^2^*p* < 0.01; ^3^*p* < 0.001, significant difference compared to sham-operated control; ^c^*p* < 0.001, significant difference compared to Ovx control. PS = *P*. *soyauxii*.

According to Figure 3, the histomorphometry of vaginal histological sections showed a significant (*p* < 0.001) increase in the size of the vaginal epithelium at the doses of 200 and 300 mg/kg and significant decrease in the size of the vaginal epithelium at a dose of 400 mg/kg.

#### 3.4.3. Effect on Vaginal Cytology and Microarchitecture of Vagina, Uterus, and Mammary Gland

**Figure 4:**
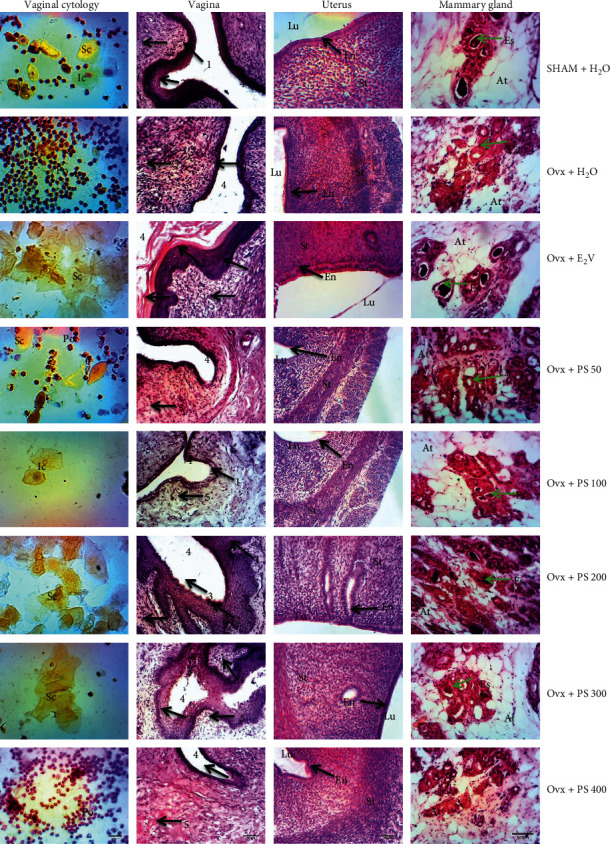
Microphotographies of vaginal cytology (100x, Papanicolaou) and the microarchitecture of vagina, uterus, and mammary gland (HE, 100x) after 3-days treatment with *Pterocarpus soyauxii* heartwood extract. PS= *P*. *soyauxii*; **vaginal cytology: Sc** = superficial cell; **Ic** = intermediate cell; **Po** = polynuclear; **Pc** = parabasal cell; **vagina**: **1** = *Stratum germinativum*; **2** = *Stratum granulosum*, **3** = *Stratum corneum*, **4** = lumen, **5** = chorion, **uterus: Lu** **=** uterine lumen; **En** = endometrium; **St** = myometrium; **mammary gland**: **At** = adipose tissue; **Es** = eosinophilic secretion; **Ca** = cell layer of the acinus.

Figure 4 represents effects of 3-day treatment with aqueous extract of *P*. *soyauxii* heartwood on the vaginal cytology and microarchitecture of vaginal, uterus, and mammary gland. The three-day treatment with the aqueous extract of *P*. *soyauxii* heartwood 14 days after ovariectomy resulted in the appearance of superficial cells on vaginal smears of female rats treated with the extract at the doses of 100, 200, 300, and 400 mg/kg as well as estradiol valerate. However, the vaginal smear of the rats treated with the extract at a dose of 50 mg/kg revealed parabasal, polynuclear, and some superficial cells, which indicate a cycle at the metestrus stage. According to Figure 4, the 3-day treatment with plant extract and E_2_V resulted in stratification of the vaginal epithelium at doses of 200 and 300 mg/kg compared with that of Ovx group animals treated with distilled water. On the mammary gland, *P*. *soyauxii* aqueous extract at doses of 100, 200, and 300 mg/kg like E_2_V compared to Ovx animals induced a differentiation of the cell layers of the acinus, an increase of eosinophilic secretions in the lumen of the acini, and heights lobule in Ovx rats. The microphotographies of uterus shows a differentiation of the endometrium with both estradiol valerate and the extract at doses of 200 and 300 mg/kg compared to the Ovx group.

### 3.5. P. soyauxii Activities in a Postmenopausal Model

#### 3.5.1. Effects on the Vaginal Epithelium

**Figure 5:**
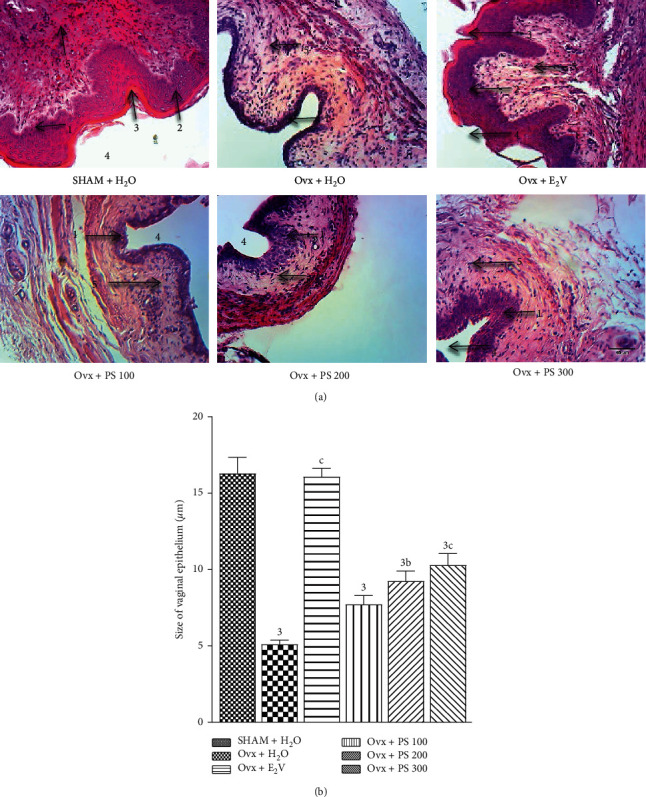
Effects of a 28-day treatment of with PS on vaginal epithelium (100x, haematoxylin-eosin). ^3^*p* < 0.001, significant difference compared to sham-operated control; ^b^*p* < 0.01; ^c^*p* < 0.001, significant difference compared to Ovx control. **1** = *Stratum germinativum*; **2** = *Stratum granulosum*, **3** = *Stratum corneum*, **4** = lumen, **5** = chorion.

According to Figure 5(a), the 28-day treatment with plant extract resulted in stratification of the vaginal epithelium at the doses of 200 and 300 mg/kg compared with that of Ovx group animals treated with distilled water. In addition, the histomorphometry of vaginal histological sections shows a significant (*p* < 0.01) increase in the height of the vaginal epithelium height doses of 200 mg/kg. The dose of 300 mg/kg induced a significant (*p* < 0.001) increase in the size of the vaginal epithelium.

#### 3.5.2. Effects on the Uterine Epithelium

**Figure 6:**
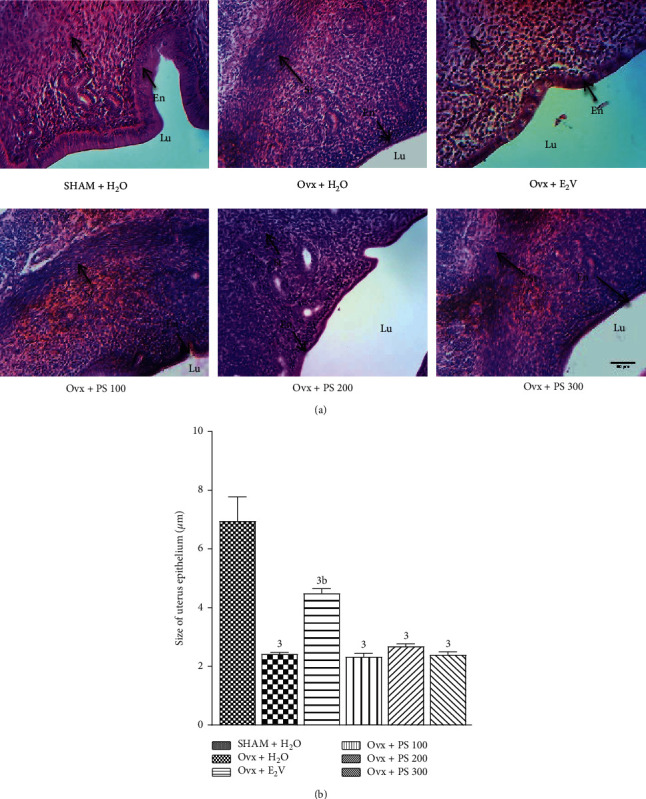
Effects of a 28-day treatment with *P*. *soyauxii* on uterine epithelium (100X, haematoxylin-eosin). ^3^*p* < 0.001, significant difference compared to sham-operated control; ^b^*p* < 0.01, significant difference compared to Ovx rats treated with distilled water. **PS** = *P*. *soyauxii*; **Lu** **=** uterine lumen; **En** = endometrium; **St** = myometrium.

The 28-day treatment with the extract of *P*. *soyauxii* did not have any effect on uterine epithelium at all doses. As shown by Figure 6, the uterine epithelium is simple cubic in all rats treated with the extract of *P*. *soyauxii* contrary to the one treated with estradiol valerate which is stratified.

#### 3.5.3. Effects on the Mammary Gland

**Figure 7:**
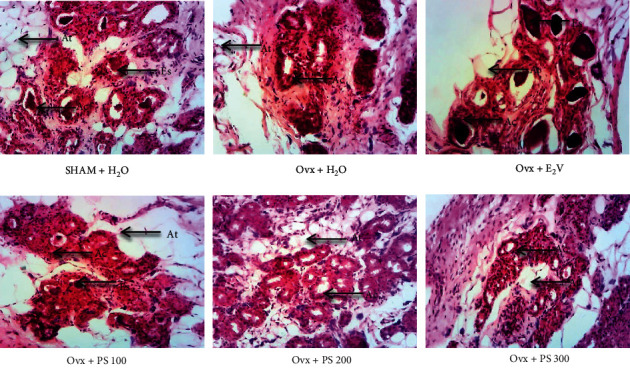
Effect of a 28-day treatment with PS on mammary gland (100×, haematoxylin-eosin) showing the differentiation in Ovx rats. **At** = adipose tissue; **Es** = eosinophilic secretion; **Ca** = cell layer of the acinus. (a) SHAM + H_2_O. (b) Ovx + H_2_O. (c) Ovx + E_2_V. (d) Ovx + PS 100. (e) Ovx + PS 200. (f) Ovx + PS 300.

**Figure 8:**
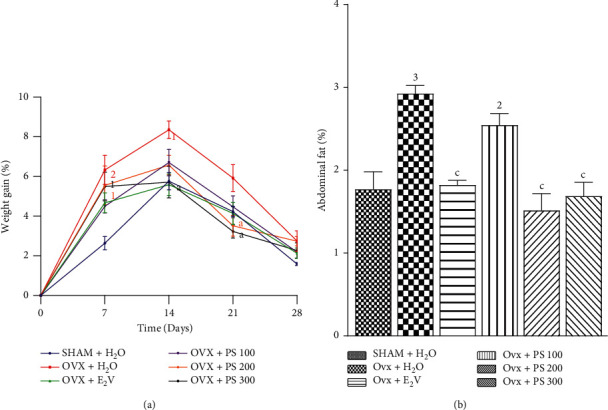
Effects of a 28-day *P*. *soyauxii* treatment on weight gain (a) and abdominal fat (b). ^1^*p* < 0.05; ^2^*p* < 0.01; ^3^*p* < 0.001, significant difference compared to sham-operated control; ^a^*p* < 0.05; ^b^*p* < 0.01; ^c^*p* < 0.001, significant difference compared to Ovx control; **PS** = *P*. *soyauxii*.

As shown by Figure 7, *P*. *soyauxii* at 100 mg/kg exhibits a cell differentiation and production of eosinophilic secretions according to Figure 8. Nevertheless, this differentiation is weaker than E_2_V one.

#### 3.5.4. Effects on Weight Gain and Abdominal Fat

Figure 8(a) shows a significant increase in weight gain on days 7 and 14 of treatment, respectively, of *p* < 0.01 and *p* < 0.05 in ovariectomized rats compared with sham-operated animals. Likewise, Figure 7(b) shows a significant increase (*p* < 0.001) in the relative weight of abdominal fat in ovariectomized rats treated with distilled water compared to sham-operated rats. The extract of *P*. *soyauxii* at the dose of 300 mg/kg induced a significant decrease (*p* < 0.05) in weight gain on 14 and 21 days of treatment and the same effect (*p* < 0.001) on relative weight abdominal fat compared to Ovx animals. Extract at the dose of 200 mg/kg decreased only relative weight abdominal fat (*p* < 0.001) and weight gain on 21 days of treatment compared to Ovx animals.

#### 3.5.5. Effects on Serum Glucose Levels during Insulin Resistance Test

**Figure 9:**
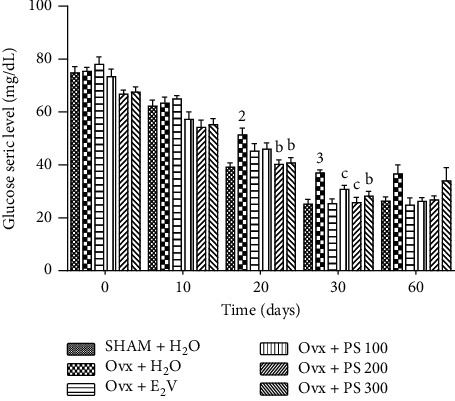
Effects of a 21-day treatment with *P. soyauxii* on serum glucose levels during insulin resistance test. ^1^*p* < 0.05; ^2^*p* < 0.01; ^3^*p* < 0.001, significant difference compared to sham-operated control; ^a^*p* < 0.05; ^b^*p* < 0.01; ^c^*p* < 0.001, significant difference compared to Ovx control; **PS** = *P*. *soyauxii*.

Figure 9 below shows a significant increase in serum glucose levels of (*p* < 0.01) and (*p* < 0.001), respectively, at 20 and 30 minutes in ovariectomized rats compared to sham-operated rats. The extract of *P*. *soyauxii* at the dose of 200 mg/kg resulted in a significant decrease in serum glucose levels at 20 and 30 minutes of *p* < 0.01 and *p* < 0.001, respectively, as compared to Ovx animals. The extract at the dose of 300 mg/kg resulted also in a significant decrease in serum glucose levels at 20 and 30 minutes of *p* < 0.05 and *p* < 0.01, respectively, as compared to Ovx animals.

#### 3.5.6. Effects on Relative Weight of the Aorta and on Aortic Protein

**Figure 10:**
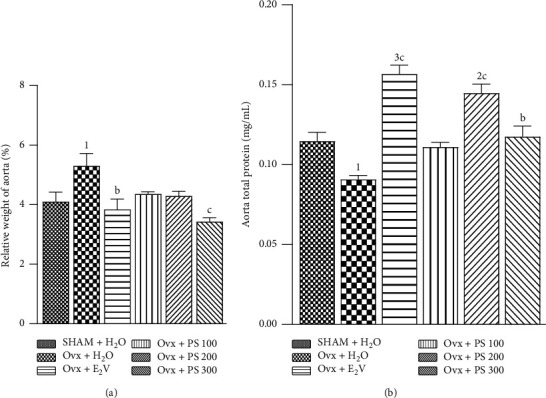
Effects of a 28-day treatment with *P. soyauxii* on fresh aorta weight (a) and aorta total protein levels (b). 1*p* < 0.05; ^2^*p* < 0.01; ^3^*p* < 0.001, significant difference compared to sham-operated control; ^b^*p* < 0.01; ^c^*p* < 0.001, significant difference compared to Ovx control.

Figure 10 shows a significant decrease (*p* < 0.05) in the aortic protein. There was also a significant (*p* < 0.05) increase in the relative weight of the aorta in Ovx rats as compared to sham-operated rats (Figure 10(a)). The extract of *P*. *soyauxii* at the dose of 300 mg/kg resulted in a significant increase (*p* < 0.001) in aortic protein levels and a significant decrease (*p* < 0.001) in relative weight of this organ. *P*. *soyauxii* extract at the doses of 100 and 200 mg/kg significantly increased protein levels (*p* < 0.001) but had no effect on relative aortic weight.

#### 3.5.7. Effects of P. soyauxii on Serum Lipid Levels

Ovariectomy caused a substantial increase in the TC, TG, LDL-C, and VLDL-C levels (Table 4) and also caused substantial decrease in HDL-C levels and increase in atherogenic index as compared to sham-operated control group (*p* < 0.001). In Ovx rats, treatment with 100 mg/kg/day of *P*. *soyauxii* induced a significant decrease in TG, LDL-C, and VLDL-C levels and atherogenic index (*p* < 0.05, *p* < 0.001, *p* < 0.001, and *p* < 0.05) but did not affect HDL-C and TC levels compared with the Ovx-control group. *P*. *soyauxii* at the dose of 200 mg/kg/day has significantly reduced TC, TG, LDL-C, and VLDL-C levels and atherogenic index (*p* < 0.01, *p* < 0.001, *p* < 0.001, *p* < 0.001 and *p* < 0.001) and increased HDL-C levels (*p* < 0.05) as compared to Ovx-control group. At the dose of 300 mg/kg/day, *P*. *soyauxii* extract significantly decreased TG, LDL-C, and VLDL-C levels and atherogenic index (*p* < 0.001, *p* < 0.001, *p* < 0.001, and *p* < 0.001) and significantly increased HDL-C levels (*p* < 0.05) as compared to Ovx group.

#### 3.5.8. Effects of P. soyauxii on Oxidative Stress Status

**Figure 11:**
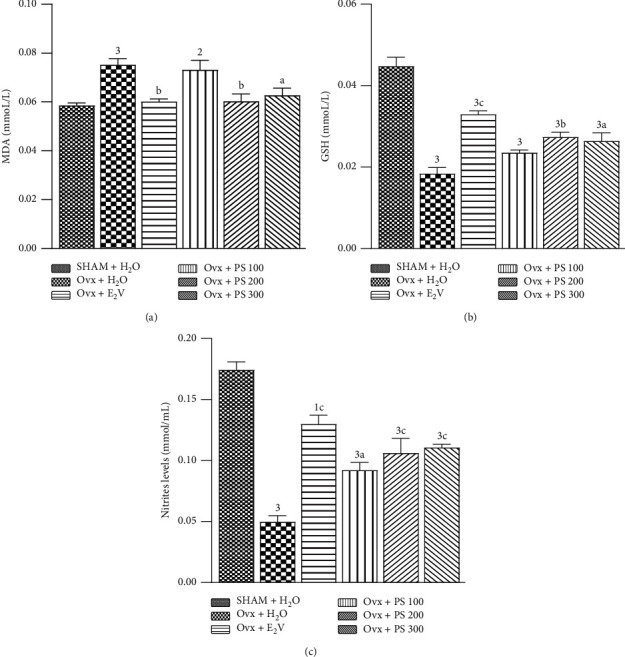
Effects of a 28-day treatment with *P*. *soyauxii* on MDA (a), GSH (b), and nitrites (c) aorta levels. ^1^*p* < 0.05; ^2^*p* < 0.01; ^3^*p* < 0.001, significant difference compared to sham-operated control; ^a^*p* < 0.05; ^b^*p* < 0.01; ^c^*p* < 0.001, significant difference compared to Ovx control; **PS** = *P*. *soyauxii*; **MDA** = Malondialdehyde; **GSH** = reduced glutathione.

After 84 days, ovariectomy resulted in a significant increase (*p* < 0.001) in MDA level and a significant decrease (*p* < 0.001) in GSH and nitrite levels in the aorta. The plant at the dose of 100 mg/kg significantly increases the levels of nitrites but had no effect on the level of GSH and MDA. *P*. *soyauxii* extract at the dose of 200 and 300 mg/kg significantly decreased (*p* < 0.001, *p* < 0.01, resp.) the MDA level and likewise increased (*p* < 0.001, *p* < 0.01, resp.) GSH and nitrites levels (Figure 11).

#### 3.5.9. Effects of P. soyauxii on Aorta Histology

**Figure 12:**
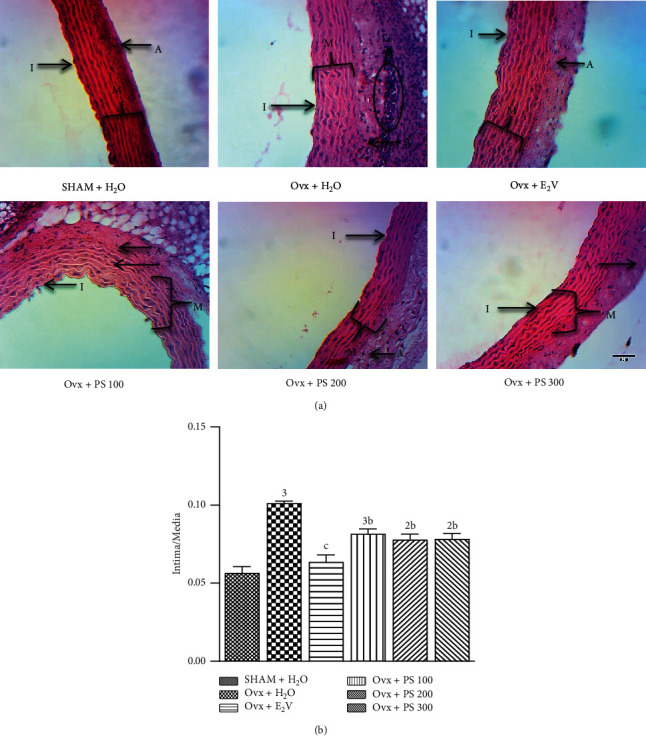
Effects of *P*. *soyauxii* 28-day treatment on aorta (100×, haematoxylin-eosin). **PS** = *P*. *soyauxii*; (I) intima; (M): media; (A): adventitia; **Li** = leukocyte infiltration.

As shown in Figure 12, after 84 days, ovariectomy induced leukocyte infiltration in aorta compared to sham-operated rats. Leucocyte infiltration was associated with a high ratio intima/media (*p* < 0.001) in Ovx rats. The extract at the dose of 100, 200, and 300 mg/kg prevented leukocyte infiltration on aorta sections. Furthermore, *P*. *soyauxii* extract significantly reduced at all doses the ratio intima/media in Ovx rats compared to the control group treated with distilled water.

## 4. Discussion

The aim of the present study was to assess estrogenic and antioxidant activities of aqueous extract of *P*. *soyauxii* heartwood in a model of oophorectomy in Wistar rats. For this, a 3-day uterotrophic assay according to the protocol of the Organization for Economic Cooperation and development [26] was used to verify estrogenic potential of the extract in ovariectomized rats. *In vitro* antioxidant capacity of *P*. *soyauxii* heartwood was evaluated using protocols described by numerous authors [23–25]. Then, an 84-day postoophorectomy model was used as described in numerous studies [13, 14] to assess properties of the plant extract on postmenopausal cardiovascular disorders and aortic oxidative stress. Compared to Ovx control, results of the 3-day uterotrophic assay showed that aqueous extract of *P*. *soyauxii* heartwood at 200 and 300 mg/kg exhibited uterotrophic activities characterized by an increase of relative weight, epithelial height, and protein level of the uterus. Plant extract also leaded to vagina cornification and stratification correlated with an increase of vaginal epithelium height and the density of foliaceous cells. The uterotrophic assay also showed an increased eosinophilic secretion in lumen of mammary acini at 200 and 300 mg/kg. Indeed, it is histologically known that oophorectomy leads to atrophy of estrogen-dependent tissues like a decrease of vaginal and uterine epithelia heights; it also reduced eosinophilic secretions in the lumen of mammary acini [33–35]. Estrogenic activities of *P*. *soyauxii* extract could be due to flavonoids found in the plant extract (63.42 ± 2.16 quercetin equivalent). As a matter of fact, flavonoids represent the most potent phytoestrogen family [36]. The results of *in vitro* antioxidant assay showed that *P*. *soyauxii* extract has induced inhibition of the DPPH and ABTS free radical with IC_50_ of 730.20 and 892.90 *µ*g/mL, respectively. This antioxidant activity could be linked to the richness of the plant extract in phenolic compounds and linoleic acid according to LC-MS analysis. Indeed, linoleic acid can prevent the production of ROS by protecting membranes composed of 1-palmitoyl 2-linoleoyl phosphatidylcholine [37]. Besides, purslane leaves like *P*. *soyauxii* aqueous extract exhibit antioxidant-like properties due to phenols and linoleic acid [38].

These estrogenic and antioxidant activities of *P*. *soyauxii* extract can be beneficial for deep hypoestrogenism observed in postmenopausal women. Indeed, oophorectomy leads to a reduction in circulating estrogen levels [39]. In rats, long-term oophorectomy is an appropriate experimental research model to study postmenopausal disorders [40] such as atrophy of estrogen-dependent tissues, cardiovascular risk factors primarily obesity, and dyslipidaemia [41, 42]. In this study, compared to sham-operated group, 84-days oophorectomy resulted in an atrophy of estrogen-dependent tissues characterized firstly by a decrease of uterus relative weight, epithelial height, and protein level, and secondly by a decrease of vagina epithelial height as well as a decrease of mammary acini diameter and a lack of eosinophilic secretions in acini lumen. These effects could be attributed to ovarian loss. Indeed, ovaries estradiol normally supplies dynamic development of estrogen-dependent tissues via a proliferative activity involving estrogen receptors [43]. The 28-days treatment with *Pterocarpus soyauxii* aqueous extract resulted in stratification and an increase of vagina epithelial height without noticeable effects on mammary gland and uterus. Effect on vagina after 28-days treatment could be due to linoleic acid, known as promoting vaginal cornification [44]. This lack of long-term action of the plant extract on mammary gland and uterus could reflect a potential selective effect, useful for preventing estrogen-dependent cancers. In addition, LC-MS analysis revealed the presence in the plant extract of mono(2-ethylhexyl) phthalate (MEHP) and 3′,5′-dimethoxy-4-Stilbenol or pterostilbene which are known as selective estrogen receptor modulators (SERM). This differing agonist or antagonist effects at the estrogen receptor in different tissues of pterostilbene contained in the plant extract can prevent hormone responsive cancers [45, 46].

In addition to genital atrophy, oophorectomy resulted in an increase of relative weight of abdominal fat, weight gain, and insulin resistance correlated with dyslipidaemia and an increased atherogenic index, similar to studies carried by Somayeh et al. [47] and Dzeufiet et al. [11]. Indeed, estrogen deficiency increases lipoprotein lipase activity, leading to blood fatty acids accumulation, and thus dyslipidaemia [48]. According to Wade [49], estrogen negatively regulates food intake and weight gain. Thereby, increased body weight and abdominal fat in ovariectomized rats in this study could be due to high food intake and fat accumulation in adipose tissue [50]. It has also been established that free radical oxidation of LDL cholesterol leads to atherosclerosis [11]. In the present study, the increase of atherogenic index is associated with oxidative stress in ovariectomized rats. Indeed, in addition to an increased LDL cholesterol, ovariectomized rats exhibited a high lipid peroxidation via an increase of MDA level. They showed also a decrease of GSH concentration which characterized an oxidative stress. Compared to Ovx control, *P*. *soyauxii* extract significantly reduced abdominal fat weight and body weights, insulin resistance, and dyslipidaemia. These effects could be due to the 3′,5′-dimethoxy-4-stilbenol also called pterostilbene which was revealed by LC-MS analysis. Indeed, pterostilbene is known for its antiadipogenic and hypotriglyceridemic properties through inhibition of proliferation and differentiation of 3T3-L1 cells into adipocytes, fatty acids accumulation, and expression of Diacylglycerol O-acyltransferase 1 (DGAT1) which supply triglycerides synthesis. In addition, pterostilbene is known to reduce expression of Peroxisome Proliferator Activated Receptors *γ* (PPAR*γ*) involved in the starting of insulin resistance and dyslipidaemia [51, 52].

Oophorectomy is associated with a high activity of nicotinamide adenine dinucleotide phosphate (NADPH) oxidase, leading to the formation of free radicals in the mitochondria. The high production of free radicals is a major contributor in the pathogenesis of atherosclerosis through LDL cholesterol oxidation [53], starting process of endothelial dysfunction leading to a decreased nitrogen monoxide levels in aorta. This atherogenic process constitutes an inflammatory state materialized in this work by leukocyte infiltration on aortic sections in Ovx control. The treatment with plant extract improved the oxidative status as well as leukocyte infiltration in the aorta at 100, 200, and 300 mg/kg. Indeed, pterostilbene is known to exert antioxidant and anti-inflammatory activities. They decreased expression of NADPH oxidase and inactivation of NF-*κ*B via a downregulation of Toll-like 5 receptors [54].

## 5. Conclusions

Sixteen-week bilateral oophorectomy induced postmenopausal symptoms in rats like vagina atrophy, dyslipidaemia, insulin resistance, weight gain, and oxidative stress. *P*. *soyauxii* aqueous extract contains mono(2-ethylhexyl) phthalate, cembrene, 3′,5′-dimethoxy-4-stilbenol, and linoleic acid. Furthermore, this extract is able to exhibit estrogenic and antioxidant activities which are probably responsible of the prevention of postmenopausal symptoms. There is a need to evaluate different pathways involved by secondary metabolites of this extract on postmenopausal symptoms.

## Figures and Tables

**Table 1:** Phytochemical analysis of *P*. *soyauxii* aqueous extract.

Secondary metabolites	Aqueous extract of *Pterocarpus soyauxii* heartwood
Flavonoids (mg EQ/g)	63.42 ± 2.16
Polyphenols (mg EAG/g)	229.42 ± 3.62
Tannins (mg ETA/g)	27.88 ± 0.23

mg ETA/*g* = mg equivalent of tannic acid per gram of extract. mg EGA/*g* = mg equivalent of gallic acid per gram of extract. mg EQ/*g* = mg equivalent of quercetin per gram of extract.

**Table 2:** Main signals exhibited in the LC-MS spectra of compounds detected in *Pterocarpus soyauxii* and proposed attribution.

N°	Tr (min)	[*M* + H]^+^		Molecular formula	Name of compound	Molecular classes
		Exp.	Calcl.			
1	0.4	235.2035	235.2056	C_16_H_27_O	Ambrial	—
2	0.5	215.0544	215.0550	C_9_H_11_O_6_	Not identified	—
3	0.6	311.0875	311.0914	C_18_H_15_O_5_	7-O-Acetylformononetin	Isoflavone
4	0.7	301.0738	301.0707	C_16_ H_13_O_6_	Khrinone A	Isoflavonoid
5	0.8	181.1063	181.1071	C_7_H_17_O_5_	Not identified	—
6	2.4	195.1223	195.1227	C_8_H_19_O_5_	Ammoniated polyethylene glycol	—
7	2.6	279.1609	279.1591	C_16_ H_23_O_4_	Mono(2-ethylhexyl) phthalate	Aromatic dicarboxylic acid
8	2.8	437.2909	437.2898	C_25_H_41_O_6_	Not identified	—
9	2.9	273.2577	273.2577	C_20_ H_33_	Cembrene A	Diterpene
10	2.9	305.1074	305.1078	C_9_H_21_O_11_	Not identified	—
11	3.0	209.1507	209.1536	C_13_H_21_O_2_	Not identified	—
12	3.1	211.1301	211.1329	C_12_H_19_O_3_	Not identified	—
13	3.2	293.2127	293.2111	C_18_H_29_O_3_	Not identified	—
14	3.5	257.1210	257.1172	C_16_H_17_O_3_	3′,5′-Dimethoxy-4-stilbenol	Stilbenoid
15	3.8	281.2495	281.2474	C_18_H_33_O_2_	Linoleic acid	Fatty acid
16	4.0	235.2054	235.2056	C_16_ H_27_ O	Not identified	—
17	4.1	263.2378	263.2369	C_18_H_31_O	Not identified	—

**Table 3:** Inhibitory concentration 50 of *P. soyauxii* aqueous extract against DPPH and ABTS radicals and FRAP concentration.

Parameters substances	FRAP (mEAG/g)	IC_50_ of DPPH (*µ*g/mL)	IC_50_ of ABTS (*µ*g/mL)
Vitamin C	—	24.56	37.75
PS extract	765.79	730.20	892.90

**Table 4:** Effects of a 28-day treatment with *P. soyauxii* on lipid profile.

Groups parameters	Sham + H_2_O	OVX + H_2_O	OVX + E_2_V	OVX + PS 100	OVX + PS 200	OVX + PS 300
TC (mmol/L)	1.43 ± 0.03	1.85 ± 0.03^3^	1.56 ± 0.05^c^	1.80 ± 0.03^3^	1.57 ± 0.01^b^	1.69 ± 0.02
HDL-C (mmol/L)	0.97 ± 0.02	0.55 ± 0.01^3^	0.78 ± 0.04^1b^	0.54 ± 0.02^3^	0.74 ± 0.02^2a^	0.74 ± 0.01^2a^
LDL-C (mmol/L)	0.32 ± 0.05	1.12 ± 0.02^3^	0.56 ± 0.07^c^	0.61 ± 0.06^c^	0.71 ± 0.03^3c^	0.60 ± 0.06^c^
TG (mmol/L)	0.63 ± 0.02	0.84 ± 0.00^3^	0.68 ± 0.02^b^	0.73 ± 0.03	0.67 ± 0.00^b^	0.69 ± 0.01^b^
VLDL (mmol/L)	0.12 ± 0.00	0.16 ± 0.00^3^	0.12 ± 0.00^c^	0.14 ± 0.00	0.13 ± 0.00^b^	0.14 ± 0.00^c^
AI	1.45 ± 0.05	3.31 ± 0.05^3^	2.20 ± 0.12^3c^	3.44 ± 0.06	2.24 ± 0.08^c^	2.17 ± 0.13^3c^

Values represent means ± SEM (*n* = 5); ^1^*p* < 0.05; ^2^*p* < 0.01; ^3^*p* < 0.001, significant difference compared to sham-operated control; ^a^*p* <0.05; ^b^*p* < 0.01; ^c^*p* < 0.001, significant difference compared to Ovx control; **PS** = *P*. *soyauxii*.

## Data Availability

The data are available upon request.

## Abbreviation

ABTS: 2,2′-Azinobis-3- ethylbenzothiazoline-6-sulfonic acid
AI: Atherogenic index
DAD: Diode array detector
DGAT1: Diacylglycerol O-acyltransferase 1
DPPH: 1,1-Diphenyl-2-picrylhydrazyl
E_2_V: Estradiol valerate
FRAP: Ferric-reducing antioxidant power
GSH: Reduced glutathione
HED: Human equivalent dose
HDL-C: High-density lipoprotein cholesterol
HRT: Hormone replacement therapy
IC50: Inhibitory concentration 50
ITT: Insulin tolerance test
LC-MS: Liquid chromatography-mass spectrometry
LDL-C: Low-density lipoprotein cholesterol
MDA: Malondialdehyde
MEHP: Mono(2-ethylhexyl) phthalate
NF-*κ*B: Nuclear factor kappa-light-chain-enhancer of activated B cells
Ovx: Ovariectomized animals
PPAR*γ*: Peroxisome proliferator activated receptors *γ*
PS: Pterocarpus soyauxii
ROS: Reactive oxygen species
SERM: Selective estrogen receptor modulators
TC: Total cholesterol
TG: Triglycerides
TPTZ: 2,4,6-Tri(2-pyridyl)-s-triazine
UHPLC-MS: Ultra-High-Performance Liquid Chromatography tandem mass spectrometry
VLDL-C: Very-low-density lipoprotein cholesterol.
